# Cold Atmospheric Plasma: A Powerful Tool for Modern Medicine

**DOI:** 10.3390/ijms21082932

**Published:** 2020-04-22

**Authors:** Dušan Braný, Dana Dvorská, Erika Halašová, Henrieta Škovierová

**Affiliations:** Biomedical Center Martin, Jessenius Faculty of Medicine in Martin, Comenius University in Bratislava, 036 01 Martin, Slovakia; dusan.brany@uniba.sk (D.B.); erika.halasova@uniba.sk (E.H.); henrieta.skovierova@uniba.sk (H.Š.)

**Keywords:** cold atmospheric plasma, wound healing, oncology, regenerative medicine, plasma

## Abstract

Cold atmospheric plasma use in clinical studies is mainly limited to the treatment of chronic wounds, but its application in a wide range of medical fields is now the goal of many analyses. It is therefore likely that its application spectrum will be expanded in the future. Cold atmospheric plasma has been shown to reduce microbial load without any known significant negative effects on healthy tissues, and this should enhance its possible application to any microbial infection site. It has also been shown to have anti-tumour effects. In addition, it acts proliferatively on stem cells and other cultivated cells, and the highly increased nitric oxide levels have a very important effect on this proliferation. Cold atmospheric plasma use may also have a beneficial effect on immunotherapy in cancer patients. Finally, it is possible that the use of plasma devices will not remain limited to surface structures, because current endeavours to develop sufficiently miniature microplasma devices could very likely lead to its application in subcutaneous and internal structures. This study summarises the available literature on cold plasma action mechanisms and analyses of its current in vivo and in vitro use, primarily in the fields of regenerative and dental medicine and oncology.

## 1. Introduction

William Crookes established the fundamentals of plasma science in 1879 by experimentally ionizing gas in an electrical discharge tube by the application of high voltage through a voltage coil. The ionised gas was named radiant matter. The current plasma term was then initiated almost fifty years later in 1927 by Irvin Langmuir [1]. Plasma has since been applied in many spheres over the past few decades, including medicine [2,3,4]. Plasma is often defined as an ionised gas produced by disintegration of polyatomic gas molecules or the removal of electrons from monatomic gas shells [5]. However, not every ionised gas that contains charged particles can be considered plasma because of the following strict definition [5,6,7]: (1) Plasma must have macromolecular neutrality (quasi-neutrality). In the absence of external disturbances in plasma, the net resulting electric charge is zero. Therefore, plasma contains (almost) the same density of positively and negatively charged particles; (2) Plasma must have Debye shielding, where the charged particles in plasma are arranged to effectively shield electrostatic fields within the distance of a Debye length. This is defined as a measure of the distance over which the influence of the electric field of an individual charged particle is felt by other charged particles inside the plasma; (3) Plasma frequency. If plasma loses its equilibrium conditions, the resulting internal space charge-fields promote collective particle motion that tends to restore the original charge neutrality. This motion is characterised by the natural oscillation frequency referred to as plasma frequency. Plasma can therefore be defined as “a quasineutral gas containing many interacting free electrons and ionised atoms and molecules which have collective behavior caused by long-range coulomb forces” [7]. In addition, the charged-particle motion in plasma gives rise to electric fields and generates currents and magnetic fields [6].

Plasma can also be divided into high-temperature, thermal, and non-thermal groups [7,8]. All particles (electrons and heavy particles) have the same temperature in high temperature plasma, and they are therefore in thermal equilibrium. In thermal (quasi-equilibrium plasma) are only areas of thermal equilibrium within the plasma. Finally, non-thermal (non-equilibrium) plasma have particles that are not in thermal equilibrium. This plasma is termed “cold plasma” [7,8].

High-temperature or thermal plasmas have higher electron density and ionization than cold plasma, which has ionization only up to 1% [5,6,7]. In poorly ionized plasmas, the charge-neutral interactions dominate multiple coulomb interactions [6]. Moreover, although electron–electron collisions achieve thermodynamic equilibrium in cold plasma, and their temperature is then much higher than that of ions and neutrons, they cannot transfer their kinetic energy to larger particles [6].

High temperature plasma can reach 10^8^ K, as found in the solar core. Thermal plasma temperature can be approximately 2 × 10^4^ K and this is observed in thunderstorm lightning. Finally, nonthermal plasma temperature can be 300 to 1000 K under artificially created conditions, as in fluorescent tubes [8,9]. Cold plasma discharge can be achieved at both low pressure and atmospheric pressure [2,3,10].

Low pressure cold plasma was first applied in the late 1960s to decontaminate surfaces, and this treatment has in some aspects proven to be more effective than conventional sterilisation [11]. In contrast, the benefits of cold atmospheric plasma (CAP) in reducing microbial load were discovered only in the second half of the 1990s [12,13]. Nevertheless, CAP provides a better alternative for modern medicine than the cold plasma generated at low pressure. CAP application is also an easier process to use than low pressure plasma because it can be generated from a portable device and this enables easier access to affected cells and tissues than the that of the large vacuum generating system required for low pressure cold plasma. In addition, most types of low-pressure plasma generating systems are very expensive [11]. It is also impractical, and almost impossible, to place any body part in this system and leave it to be exposed to plasma discharge. Moreover, all animal and human tissues contain water and its presence is undesirable in low pressure conditions [11]. Finally, although low-pressure plasma discharge can be regulated, its character is quite different and stronger than the discharge generated by atmospheric pressure plasma and is generally not suitable for application to human or animal cells or tissues. Nevertheless, cold plasma generated under low pressure can be utilised in medicine. Examples of this are its use in “flash-sterilisation”, implants, and tissue engineering products’ surface modifications [11].

Although CAP does not achieve the sterilization and decontamination possibilities of low pressure plasma [11], it is still effective in lowering microbial load [14,15]. Most importantly, CAP’s less intense effects enable its direct application to cells and tissues [3,4] and its ability to reduce microbial load makes it a good option to replace antibiotics and to combat bacterial strains that have increasing antibiotic resistance [3]. A further great advantage of CAP-generating devices is their relatively low manufacturing costs [3,4]. Therefore, accessible, efficient, and cheaper CAP devices can very likely reduce the financial burden imposed on the health budget by conventional treatments.

Plasma discharge reduces bacteria viability mainly by formation of UV radiation, induction of reactive oxygen (ROS) and nitrogen (RNS) species, and creation of electric current [4,16,17,18,19,20,21,22]. The oxygen-based species generated by plasma discharge include hydroxyl (OH), hydrogen peroxide (H_2_O_2_), superoxide (O_2_^−^•), hydroxyl radical (•OH), singlet oxygen (^1^O_2_), and ozone (O_3_) [23,24]. The nitrogen-based species are nitric oxide (•NO), nitrogen dioxide (•NO_2_), dinitrogen tetroxide (N_2_O_4_), nitrogen trioxide (NO_3_), nitrous oxide (N_2_O), and peroxynitrite (ONOO-) [23,24]. The reactive species can be formed either by plasma–air interaction or by plasma–liquid interaction. While hydroxyl radicals and nitric oxides are typically formed by plasma–air interactions, nitrites, nitrates, and H_2_O_2_ with relatively longer lifetimes are formed by plasma–liquid interactions [23,24]. For example, this latter interaction occurs when cells in the cultivation media are exposed to plasma [25]. In addition, CAP generates positively charged ions such as N^2+^ [22] and also electrons [20].

However, prokaryotic bacterial cells differ from eukaryotic human and animal cells, and tumour cells differ from healthy cells. Moreover, all cells can act differently in in vitro conditions and they can respond differently to the cells in living organism tissues. This highlights the possibility of different responses to CAP devices in those cell types and also under in vitro and in vivo conditions [26]. However, it is generally presumed that low dose plasma treatment stimulates cell viability and enhances proliferation, differentiation, and migration, while high doses should induce cell apoptosis [26].

Plasma devices used in clinical practice are designed to avoid danger to healthy cells. They undergo multistep testing and are not currently associated with significant side effects [4,27,28]. However, the appropriate CAP dosage in clinical practice must be closely controlled, and this control depends on treatment type. For example, the distance between the plasma source and treatment object must be carefully adjusted. Here, Nastuda et al. [29] reported that the effect of plasma jet application can vary widely, even with a one centimetre difference between tube nozzle and human skin. The authors found significant differences in plasma spread and current flow through human tissue when the skin–nozzle gap was reduced from 15 mm to 5 mm. Moreover, it cannot be hypothetically excluded that CAP application does not have some minimal negative effects at the molecular level. All these findings and possibilities are subject to ongoing analysis, but current results suggest that the unproven adverse effects are outweighed by CAP’s many benefits [3,4,12]. Finally, it is likely that more possibilities and greater benefits for CAP use in clinical practice will be demonstrated in the future, because most CAP studies have just taken place in the last 15 years [3,4,12]. While CAP can be employed in aesthetic procedures [30,31,32], herein we summarise CAP use in more serious medical conditions. These especially include acute and chronic wounds and oral bacterial infections and tumour therapy. Concurrently, we explore plasma potential in regenerative medicine and biological engineering.

The clinically used and experimentally tested CAP devices are divided into three main categories: 1.) those based on direct-discharge, 2.) those based on indirect-discharge, and 3.) hybrid types [4,33,34].

Direct dielectric barrier discharge (DBD) occurs between a high voltage electrode and a grounded one. The electrodes can both or individually be covered with a dielectric layer or the dielectric material can be placed in the space between the electrodes. DBD devices must also be charged only with alternating or pulsed high voltage to ensure capacitive coupling. Electrons and ions in these devices after initial gas breakdown are “stopped” by the dielectric barrier. Electric field of ions and electrons then shields the electric field from the external source. If this state is maintained, it leads to discharge loss, and the external voltage must be changed to preserve discharge. Breakdown occurs when increasing alternating current voltage is applied, and discharge activity ceases when the maximum voltage is attained. Moreover, two breakdowns occur in the same period when pulse-operated DBDs are employed. The initial breakdown is caused by the high voltage pulse, and the electric field created by the charge carrier is sufficient to cause a further breakdown during voltage decrease. This is in the form of a ‘backward discharge’ [35,36,37]. The DBD devices have several variations and designs, and one of the most practical and suitable for medical application is the floating electrode DBD (FE-DBD). Technically, FE-DBD devices do not contain a grounded electrode, and this is replaced by affected cells or tissues. The DBD devices provide a higher intensity and more adaptable and controlled discharge. They can also generate plasma solely in air without the need for carrier gases. The discharge area, however, is relatively limited because it must lie between two electrodes, and a constant distance must be maintained; this demands a smooth, flat surface [4,33,34].

Indirect discharge is generated by devices usually called Plasma jets, Plasma pens, or Plasma torches. These devices are similar to indirect discharge devices in that they comprise two facing electrodes generating a plasma discharge between them, but here, the carrier gas directs the plasma discharge. Therefore, the discharge does not affect the object between the two electrodes but proceeds in the gas flow direction. While this allows the target object to be located outside the device and the affected area is adjustable, lower amounts of ROS and RNS species are produced and the discharge is harder to control than with the DBD devices. Finally, plasma generated by indirect devices is stronger in all UV ranges, but unlike DBD devices, they do not produce electric current.

The hybrid plasma devices combine these principles but are currently applied only at the experimental level. These devices create a discharge by combining micro-discharges on a grounded mesh electrode. There is uniform discharge, no effect on the object between the two electrodes, and the device is relatively easy to control. However, these devices have slightly higher susceptibility to component wear and subsequent deterioration. This is especially noticeable in humid environments and following direct contact with treated cells and tissues [4,33,34].

There are three specific types of plasma devices certified for clinical practice [11]. The very first certified device was the kINPen^®^ MED plasma-pen (INP Greifswald/neoplas tools GmbH, Greifswald, Germany). The second was the PlasmaDerm^®^ VU-2010 DBD device, based on dielectric plasma discharge technology (CINOGY Technologies GmbH, Duderstadt, Germany) and the latest certified device is the SteriPlas plasma torch device (Adtec Ltd., London, UK). However, several more devices with various modifications have been tested under laboratory and experimental conditions and are awaiting possible certification for clinical application.

Finally, plasma application itself can be direct and indirect. In direct plasma application, cell lines are exposed to plasma discharges in vitro, and animal models and human tissues are exposed to the discharges in vivo. In contrast, the medium or solution affected or activated by plasma is used in indirect application. This is then used in cell cultivation or for direct injection into tested subjects, such as mouse xenografts [38]. In addition, long-term species including nitrates, nitrites, and H_2_O_2_ are usually preserved in plasma-activated medium (PAM) [39].

## 2. Use of Cold Plasma for Treatment of Chronic and Acute Wounds

The antimicrobial effect of CAP was demonstrated in the 1990s and this led to its use in medicine [12]. The initial 2007 clinical trial used the plasma device in facial rejuvenation procedures [40]. CAP use in regenerative medicine was initially aimed at accelerating acute and chronic wound healing by alleviating bacterial infection, because infection can significantly slow the healing process [4,10,27]. The first randomised pilot trials were performed by Isbary et al. [41]. These authors investigated CAP’s effect on microbial infection mitigation in chronic ulcer wounds and confirmed significant infection reduction without side effects. Two years later, they reported that two-minute CAP treatments sufficiently decreased microbial load and improved chronic ulcer healing [42]. Both studies included venous, arterial, diabetic, and traumatic ulcers, and reduced bacterial infection was observed regardless of bacterial type. Chronic leg ulcers are relatively common, especially venous ulcers that affect up to 2% of the global population [43,44]. Ulcer reduction and cure is complex and it requires long-term care. Moreover, 15% of patients have ulcers that never heal and up to 71% have complicated remission [43,44]. Bacterial infection is a major contributor to the complex treatment of ulcers and many strains present in ulcers have increasing resistance to conventional antibacterial treatment [45,46]. Studies, therefore, focused on cold plasma use in ulcer treatment. One randomised study on CAP treatment included 14 patients [47]. Half the patients were treated with conventional procedures and the remainder had concurrent CAP therapy. Treatments were administered tri-weekly for eight weeks to both groups, followed by a four-week observation period. Although reduced arterial ulcers were observed in both groups, the CAP group had more rapid and observable improvement. The effect of CAP on 50 patients with pressure ulcers was then analysed in a similarly conceived study [48]. Subjects were divided into standard treatment and combined CAP-intervention groups, and the treatments were applied weekly for eight weeks. Improvement, including bacterial load decrease, was already significantly greater in the CAP group after the first week.

CAP benefits in pressure ulcer reduction have also been recorded in Wistar rat models. Experimentally created ulcers were treated with CAP for 60 s, 3 times a day for 5 days [49]. There was resultant rapid re-epithelisation, angiogenesis, collagen synthesis, and increased tissue mechanical strength after plasma exposure. Guo and colleagues [50] investigated CAP effects on various chronic wounds, including pyoderma gangrenosum, giant genital warts, and diabetic foot ulcers. The pyoderma gangrenosum patient had previously been unsuccessfully treated with antibiotics. The lesions were irradiated for 5 min once every 2 days, with 60–80 min total irradiation for all lesions. Exudation was significantly lower on the third day, and 6 cycles of exposure enabled the lesions to completely dry and contract. This patient was treated for a further 6 months without remission. A second patient had experienced some improvement of pyoderma gangrenosum after antibiotic treatment and was then treated with this CAP regime. The lesion completely disappeared after 8 CAP treatments. There was again no recurrence noted in the 4-month follow-up period. In addition, a diabetic patient who had an ulcerated foot for two months experienced healed ulcers after four repeated CAP treatments.

Finally, two independent studies reported the possibility of concurrent CAP device use with commonly used octenidine disinfectant for chronic leg ulcer treatment. The authors considered that combined use of cold plasma and this disinfection should achieve better results than separate application [51,52]. CAP applications for chronic wound healing are listed in Table 1.

The primary intention of CAP use was to provide a modern form of wound disinfection. However, CAP treatment has already proved beneficial in wound healing due to increased cutaneous microcirculation, monocyte stimulation, keratinocyte and fibroblasts proliferation, and cell migration [26,27]. Of these, the keratinocytes and fibroblasts are especially important in the later wound-healing phases [26].

The positive effect of CAP application on keratinocyte and fibroblast proliferation has been demonstrated on cell lines in vitro [53]. The HAcaT keratinocyte cell lines and MRC5 fibroblast cell lines had increased mobility even after brief CAP exposure. This involved decreased gap-junction protein activity at the molecular level, and changes in adherent junctions and cytoskeletal dynamics with concurrent E-cadherin and integrins down-regulation. The in vivo plasma effect in mouse models was also investigated in that study, and the authors demonstrated that the observed wound healing is due to formation and action of NO, UV radiation, ROS, and RNS [53]. Several other studies also confirmed the CAP effect on keratinocyte activity. There was simultaneously increased b1-integrin expression and decreased E-cadherin and EGFR expression in these cells following CAP exposure [54]. Moreover, keratinocytes had significantly increased levels of ROS after CAP application, which resulted in various cell adaptation mechanisms [55]. More than 260 genes, including those coding for cytokines, growth factors, and antioxidant enzymes were differentially expressed. In addition, the HSP-27 cell protective heat-shock protein, which participates in regulating cell development and differentiation, was also highly expressed [55]. Schmidt et al. [56] indicate that the p53 cascade should be a major hub of cold plasma–cell interactions in keratinocytes. The authors also consider that the ATM and ATR redox sensors have higher activity, and that MAP kinase signalling should modulate the p53 signals.

A recent study [57] established interesting ‘cross-talk’ between CAP-affected fibroblasts and keratinocytes. A co-cultured model of these cells showed that plasma exposure initiated higher HIPPO pathway activity and that the transcriptional coactivator of this pathway YAP was significantly up-regulated. Moreover, the downstream effectors of this pathway and YAP target genes, *CTGF* and *CYR 61* [58], were more highly expressed, but this was only seen in fibroblasts. This increased expression, however, could be reversed by antioxidants application. Most importantly, increased HaCat keratinocyte cell mobility occurred only when they were incubated with CAP-treated fibroblast-conditioned medium. Finally, the authors presumed that CTGF and Cyr61 secretion from fibroblasts activates keratinocytes via paracrine signalling. Evidence for this claim includes that the HaCat lines had greater mobility after exposure to recombinant CTGF and Cyr61, even when CAP treated fibroblasts were not present.

It is interesting that cold plasma in animal mouse models accelerated wound healing in second- and third-degree burns. This was mainly due to increased angiogenesis. At the molecular level, NO formation was accelerated and the expression of the PDGFRβ and CD31 pro-angiogenic markers increased. There was also increased TGFβ1 activity and VEGFA/VEGFR2 signalling stimulation [59,60,61]. The positive CAP effect was also proven in rat model healing of chemical burns following rat-skin exposure to sulphuric acid. Here, 40 s exposure to CAP each day accelerated healing. The CAP treated wounds almost disappeared after 21 days, while untreated wounds remained clearly visible. The authors also noted that the biochemical profile was altered in CAP treated wounds. This was apparent in the changed levels of the following oxidative stress markers. The malondialdehyde levels were increased, and the reduced glutathione, glutathione peroxidase, and catalase levels were lower in the CAP group than those in unaffected controls, those treated with plasma modified polyurethane wound dressing, and those in the natural wound recovery group. The white blood cell dynamics and the complement component 3 and fibrinogen production differed in these groups during the 21 day treatment. White cell counts were generally highest in the natural wound recovery group and lowest in the CAP group. The fibrinogen and complement component 3 concentrations were similarly highest in the natural wound recovery group and lowest in the CAP treated group [62,63]. Betancourt-Angeles et al. then demonstrated that cold plasma accelerated human burn healing [64]. Pain and itching were relieved after a three-minute CAP application, and a further three-minute repeat application after 16 h produced significantly accelerated healing and new tissue formation. This was a case report study and the authors did not investigate the molecular mechanisms during or after plasma administration. Previous research, however, supports the expectation that angiogenesis and growth factor activation had occurred.

CAP effects on different acute wounds has also been investigated. One study involved patients with different sizes of lower extremity skin transplants. The patients were divided into two groups, with one group receiving a placebo and the other under-going CAP treatment. The final results showed that the CAP group patients had a much better healing course the second day after CAP application [65].

A further interesting study investigated the potential of plasma application to dog bite wounds [66]. Here, the authors analysed the effect of CAP on the following bacterial strains typically found in dog saliva: *Staphylococcus pseudintermedius*, *Staphylococcus aureus*, *Streptococcus canis*, *Pseudomonas aeruginosa*, *Pasteurella multocida*, and *Escherichia coli*. CAP exerted a strong in vitro antiproliferative effect on all these bacteria. However, some differences were noted between the strains, dependent on bacterial growth phase and treatment length. The author’s previous similar study [67] revealed that the overall CAP decontamination effect was lower than both polyhexanide-biguanide and saline lavages. These differences, however, were not statistically significant and the employed disinfectant did not affect the overall healing course. Therefore, further analysis would be beneficial to establish definitive conclusions on CAP use in treating dog bite wounds. However, it can be expected that the current potential for CAP application in these acute wounds will increase, especially when these bacterial strains’ increasing resistance complicates treatment. Another study involved healthy volunteers who underwent voluntary laser-ablative skin lesions. Their inflicted injuries were then treated with CAP application, and the authors’ planned CAP treatments for 30 s intervals over three days was sufficient to improve wound healing [68]. Further work by Nishijima et al. [69] studied cold plasma’s ability to accelerate healing in acute surface wounds created by a fractional CO_2_ laser. The standard clinical treatment for these wounds is topical ointment application, including steroids, petrolatum, basic fibroblast growth-factor sprays, and gels containing fullerene [69]. The study volunteers were divided into four groups: (1) no treatment, (2) treatment with CAP, (3) applied ointments containing betamethasone valerate, and (4) applied basic fibroblast growth factor sprays. Although no significant differences were observed in the groups’ overall wound healing, the CAP group had the most reduced skin redness and mean roughness. Gao et al. [50] investigated the effect of cold plasma on two traumatic wounds. The first patient had used inappropriate self-treatment with glucocorticoids and mupirocin, which resulted in self-inflicted secondary eczema with exudation and crusting. Treatment with CAP for 20 min every second day ceased wound exudation, and visible wound healing was visible after three consecutive treatment rounds. The second patient’s wound had been treated with antibiotics without success, and complete wound healing was observed after three consecutive rounds of CAP application. Finally, this study included a patient with a giant genital wart treated with CAP. The patient had this wart for three months during immuno-suppressive drug treatment, and this caused overall reduced immunity. The ablation site was treated with CAP for 40 min after wart removal and this was then repeated after a further three days. Complete healing was accomplished after two repetitions. CAP applications for acute wound healing are listed in Table 2.

## 3. Cold Plasma in Dental Medicine

Standard cleaning procedures and oral cavity disinfection is based on laser device use, mechanical infection removal, or using antimicrobial solutions. The first two procedures, however, may cause thermal or mechanical damage to tissues. This risk can be significantly decreased with CAP devices [70]. A further CAP advantage is that its discharge can be relatively easily applied to uneven surfaces and inaccessible oral cavity sites. The discharge from sufficiently miniature devices can also be applied directly to the dental canal [71]. Finally, in contrast to liquid antimicrobial solutions, CAP can be applied to discrete oral cavity sites, and unpleasant side effects from microbial solution use are not noted after CAP treatment [70,72].

In addition, bacterial strains often present in dental biofilm have increasing resistance. Neglecting dental biofilm removal can lead to serious diseases including aspiration pneumonia, endocarditis, and other systemic disorders [73,74]. Delben et al. [73] demonstrated that CAP application had significant antimicrobial effect on *Candida albicans* and *Staphylococcus aureus*, which are commonly present in dental biofilm. The reduction in microbial load with CAP device was also comparable to that for penicillin G or fluconazole administration. A similarly focused study confirmed the antimicrobial effect of cold plasma on a broad spectrum of bacteria in dental plaques, most importantly *Streptococcus mutans* [75]. Moreover, the resultant CAP antiproliferative in vitro effect on cultured cell lines was greater than that of standard chlorhexidine disinfectant use [75].

Several studies have also investigated the application of CAP devices to reduce microbial load in the dental canal. Here, electron microscope scanning revealed disappearance of the bacterial biofilm to 1 mm depth following 5 min ex vivo CAP application [76]. Armand et al. [77] then simulated *Enterococcus faecalis* infection in a sample of 100 extracted and disinfected teeth. This bacterium is relatively common in inflamed dental canals [78]. He/O_2_ plasma was the most effective in reducing the microbial load, as confirmed by electron microscope scanning (SEM), and He plasma had approximately the same efficacy as photodynamic therapy. However, the authors pointed out that the resulting effect was largely influenced by the shape of the dental canals. There was greater effect in the straight canals. Shahmohammadi Beni et al. [79] then provided a beneficial study on the possibilities and limitations of CAP application to the oral cavity. Therein they discussed CAP application related to oral cavity surface structures and they concentrated especially on the dispersal of OH radicals.

In addition to disinfection, CAP has other demonstrated benefits in dental medicine. Dong et al. [80] recorded that plasma has a positive effect on superficial dentine by increasing its binding strength to other dental structures. Moreover, CAP application should have a positive effect on strengthening links between fibre-reinforced posts and composite core material [81]. Cold plasma also has significant effect on titanium structures in the oral cavity. Here, CAP should increase hydrophilicity and also the roughness of titanium surfaces [82]. Both these aspects can improve cell adhesion, proliferation, and differentiation of osteoblasts, and these three factors can provide faster osteointegration [83]. Although the roughness increased by CAP application can induce greater bacteria accumulation [84], CAP’s antimicrobial ability can minimise this negative effect. These aspects make CAP devices very suitable for the treatment of peri-implant inflammation. CAP also affects zirconium structures. Yang et al. [85] recorded decreased microbial load and increased hydrophilicity after CAP application, and there was no change in zircon structure topology after CAP application. Finally, a study on the effects of the three types of devices on dental plaques provided interesting results. It was especially noted that there is no currently recognised dental plaque bacterial strain with resistance to CAP treatment [86]. Effects of CAP application to dental and oral cavity structures under in vitro and ex vivo conditions are listed in Table 3.

## 4. Use of CAP to Activate Proliferation in Stem and Progenitor Cells

It is expected that CAP can also affect stem cell proliferation, and these cells could then be used in regenerative medicine and medical engineering. However, only a few analyses have focused on this possibility. One study by Park et al. showed that CAP devices did actually induce the proliferation of stem cells derived from adipose tissue (ASC) without affecting their vital properties in any way [87]. Those authors reported that stem cell proliferation was 1.57-fold higher as early as 72 h after CAP treatment. The study also reported the influence of NO induced by CAP [87]. This was confirmed by the CAP device’s positive increase in stem cell proliferation being dramatically diminished by application of NO scavengers. In addition, when ASC cells that were not exposed to CAP were treated with the DETA-NONOate NO donor, they had a higher rate of proliferation than the control cells, but a significantly lower rate than those affected by CAP. These results indicate that increased NO concentration is a very important contributor to increased proliferation, but it is most likely not the only factor.

No cytotoxic or other negative impacts were observed in osteoprogenitor MC3T3-E1 cells following CAP application. Moreover, CAP induced accrued NO levels. This NO was introduced into the intracellular spaces, and its level in cultured cells could be controlled. There was consequent induction of early osteogenic differentiation in these conditions, and this was achieved even in the absence of pro-osteogenic growth factor [88]. A further study on CAP’s effect on osteo-differentiation addressed its impact again on MC3T3-E1 mouse osteoblast precursor lines [89]. There, the CAP stimulatory effect on osteogenic differentiation was comparable to that determined in the osteogenic differentiation medium. The authors also observed significant changes in the molecular cascades following CAP influence. Although PI3K/AKT and MAPK signalling decreased, there was increased expression of the osteogenic genes *RUNX2*, *OCN*, *COL1,* and *ALP*. Furthermore, CAP affected the dephosphorylation of FOXO1, which is important for proliferation and redox balance in osteoblasts and a very important controller of bone formation [90]. Although progenitor cell lines differ from stem cells in some respects, it cannot be discounted that this similar mechanism can also occur in stem cells when CAP treatment initiates osteo-differentiation.

The effect of CAP on human mesenchymal stem cells isolated from periodontal ligaments (hPDL-MSCs) was also analysed. Again, no negative or cytotoxic effect was observed. Furthermore, this CAP treatment inhibited hPDL-MSC migration and induced detachment without loss of overall cell viability. However, although cell viability was not affected, the authors stated that the cells had prolonged population doubling-time after CAP application. Most importantly, CAP promoted osteogenic differentiation in this instance [91].

Use of CAP devices can also initiate proliferation of hematopoietic stem cells and bone marrow-derived stem cells. This proliferation almost doubled in both these cell lines compared to untreated cells [92]. The authors also observed that expression of typical stem cell markers CD44 and CD105 was almost five-times higher in bone marrow cell lines, and that there was increased expression of *OCT4*, *SOX2*, and *NANOG* genes in both cell lines. Finally, the expression of genes that act on G1-S cell-cycle transition also increased and it can be presumed that CAP application can influence this cell cycle phase [92].

Interestingly, the CAP device effects may include initiating stem cell and biomaterial binding, in addition to its beneficial direct application to cells. For example, Alemi et al. [93] assessed whether cold plasma application improved stem cell and biomaterial scaffold adhesion in cartilage tissue engineering. They found that CAP enhanced scaffold surface hydrophilicity, and this significantly reduced the contact angle and helped initial cell binding.

Neurodegenerative diseases and traumatic central nervous system damage are currently difficult to treat. However, neural stem cells (NSC) could improve their treatment, and several studies have investigated NSC proliferation induced by CAP. They found that C17.2-NSC murine NSC proliferated and differentiated significantly faster following CAP treatment [94]. Moreover, almost 75% of neural stem cells differentiated into neuronal cell lines after exposure to CAP, and this percentage is greater than that achieved by specific growth factors. The neurons that differentiated from NSCs after CAP application had high β-tubulin III protein expression levels, and this is considered a typical marker for this cell type [95]. However, recent research indicates that high β-tubulin III expression is also typical in other stem cell types [96]. Furthermore, authors in this study observed only moderate differentiation from NSC to oligodendrocytes, and typical O4 protein marker expression [97] was only slightly higher. These same authors then found that astrocytes exhibited no disparity in differentiation between affected and nonaffected cells, and their typical GFAP protein marker was only minimally expressed [94,98]. Jang et al. [99] also showed that CAP has a positive effect on neural stem cell proliferation, and they recorded the following mechanisms: (1) The excited atomic oxygen in plasma phase initiated ROS and RNS formation, (2) these then interacted with reactive atoms in the extracellular liquid phase to form NO, which induced reversible cytochrome c oxidase inhibition in the mitochondrial complex IV. This resulted in increased mitochondrial O_2_^−^ production and finally (3) cytosolic hydrogen peroxide formed by O_2_^−^ dis-mutation acted as an intracellular messenger to specifically activate the Trk/Ras/ERK signalling pathway. Here, the authors considered that mitochondrial O_2_ and cytosolic H_2_O_2_ must act cooperatively because experimental cytosolic increase in H_2_O_2_ was not solely sufficient to initiate differentiation. However, some aspects, mainly how the phosphorylation of specific sites in tyrosine kinase receptor signalling occurred, remain unknown. Finally, the CAP induced neural differentiation had advantages over previously used neural differentiation inducers including retinoic acids and resveratrol. This CAP treatment enabled neurites to reach maximum length more rapidly, the differentiation efficiency increased, and almost 70% of differentiated neurons were identified as catecholaminergic DA neurons.

Bourdens et al. [100] provided different results to the previous studies. These authors recorded that, although human dermal fibroblast and adipose-derived stromal cells did not lose viability after 3 min CAP exposure, these cells developed a senescence phenotype associated with a glycolytic switch and increased mitochondria content. Most importantly, the authors noted that the observed proliferation arrest was accompanied by increased p53/p21 damage. Despite this, cell lines maintained some functional properties such as differentiation potential and immunomodulatory effects. Discussion of the role of senescent cells in wound healing has led authors to claim that CAP exposure aimed at provoking a small amount of transient senescence in some cells could be considered to enhance tissue regeneration. The truth of this claim, however, requires further analyses. CAP application effects on stem cells and progenitor cells under in vitro condition are listed in Table 4.

## 5. Use of CAP to Regenerate Other than Dermal Tissues

While plasma discharge treatment has focused to a great extent on dermal structures, there is also significant benefit in its application to deeply embedded organs and structures. A study on the use of CAP in the regeneration of nondermal structures assessed nasal mucosa regeneration [101]. Absence of this structure presents a serious problem, and its regeneration is complex and time-consuming. In addition, there is currently no effective way to accelerate its regeneration following severe injury or surgical intervention [101].

It was possible to observe the potential of cold plasma use on nasal mucosa regeneration under in vitro and in vivo conditions. Although the in vitro analyses were performed on BEAS-2B bronchial epithelial cell lines, these are very similar to nasal mucosa cells, and the regeneration complications are similar in severity. The CAP application to the bronchial cells increased cell proliferation and migration [101]. This migration was achieved by increased EGFR receptor activity and EMT signalling. Finally, rapid recovery of nasal mucosa was recorded in mouse models after brief in vivo CAP application [101].

Further effects of CAP devices on S9 bronchial epithelial stem cells have also been noted. CAP use provoked differences in the protein expression profile, and its long- and short-term exposure effects on cells also differed [102]. Further determination of CAP device effects on these cells included (1) induction of the Nrf2-mediated oxidative and endoplasmic reticulum stress response, (2) PPAR-alpha/RXR activation, (3) production of peroxisomes, and (4) prevention of apoptosis in the first hour after CAP administration. It is paradoxical that CAP initially acted as a stress factor on these cells, but subsequently triggered significant cell proliferation and cellular assembly and organisation.

Positive cold plasma effects have also been recognised in the regeneration of several neural cell types isolated from animal models [103]. While CAP stimulated neuronal regeneration and astrocyte growth in vitro, a further increased dosage induced loss of cell culture viability. Therefore, precise determination of the intensity and duration of CAP exposure is required for the treatment of both superficial and deeper structures.

## 6. Use of CAP for Tumour Treatment

CAP is a promising option for more effective tumour treatment. Its final effect on cancer cells is, however, quite interesting. Cancer cells produce higher amounts of ROS and RNS and although this increases their proliferative activity, even higher levels can cause apoptosis [104]. CAP then increases this to a level lethal to the cancer cell. This effect is caused by changes in their antioxidant system and double-strand breaks, but healthy cells should tolerate this increase [92]. The tumour cells also have more aquaporins [105] and this facilitates ROS and RNS penetration into the cell [106]. The degree of ROS and RNS diffusion into the cell can also be influenced by the membrane’s lipid structure [107]. Cancer cells generally have lower levels of cholesterol and this makes cells more susceptible to peroxidation [108,109]. Membrane lipid peroxidation then causes the cell membrane to form more pores so that ROS and RNS can diffuse to a greater extent [109]. A summary of the mechanisms involved indicates that various cellular responses are initiated by CAP application to tumour cells. This increase in reactive species is the main trigger that initiates tumour cell death. The specific responses here are apoptosis, growth inhibition, cell cycle arrest, DNA and mitochondrial damage, and even immunogenic cell death [110]. The actual result, however, is dose-dependent [111,112,113].

Keidar et al. performed a pilot experiment to test CAP use in tumour treatment. They initially observed that skin melanoma cell lines separated from the culture vessel after CAP application, and this led to their reduced numbers, while the healthy cell lines remained adhered [114]. The authors’ study of subcutaneous kidney tumours in mice showed that small tumours were ablated following CAP application and more advanced tumour size was reduced. Kaushik et al. [115] then demonstrated the effects of ROS on tumour cell mortality by assessing the plasma discharge effect on T98G, A549, HEK293, and MRC5 cell lines. The authors showed that the viability of HEK293 and MRC5 non-malignant cells was minimally affected compared to cancer cell lines. The ROS and H_2_O_2_ generated by plasma discharge altered the mitochondrial membrane potential. This initiated the intrinsic apoptotic pathway and resulted in increased overall pro-apoptotic gene expression and decreased anti-apoptotic gene expression. There was also a change in ERK1/2/MAPK cell signalling activity at the protein level. The resultant effect can be reversed with ROS scavengers.

Cancer cells also differ from healthy cells at the metabolic level. Here, cancer cell metabolic reprogramming assimilates simple carbons into macromolecules, and especially lipid, protein, and nucleic acid macromolecules. This results in intermediate metabolites that tumorous cells can use for growth and proliferation [116,117,118]. Xu et al. [119] studied CAP effects on altered cancer cell metabolic activity. Their KEGG and GC-TOFMS analyses highlighted that leukaemia cells had different alanine, aspartate, and glutamate metabolism. The authors also recorded reduced glutaminase activity in cancer cells after CAP application. This decreases the conversion of glutamine to glutamic acid. Deficiency of Glutamic acid, simultaneously with glutamine accumulation can lead to suppression of proliferation and even cell death in leukemia cells [119].

Studies on cell lines and animal models have repeatedly demonstrated the benefits of CAP devices and their anti-tumour activity. One study reported that glioblastoma cell lines lost their viability after plasma treatment [120]. This study also showed that the cell lines previously resistant to a temozolomide alkylating agent regained sensitivity to it [120]. In addition, several authors have reported that CAP exposure caused lost viability and induced apoptosis in other types of brain tumour cell lines [121,122,123].

Induced apoptosis has been documented in lung cancer TC-1 cell lines after plasma discharge. This has also been recorded in fibroblast lines, but to a significantly lesser extent [124]. However, the main benefit of that last study was the expressed concept of minimising the size of plasma devices to enable easier CAP use in deeper lesions and structures. Herein, apoptosis induction was triggered by plasma discharges generated from devices with 125 to 440 μm diameter. µCAP devices [125] have been used directly on mouse brain tumours and also in vitro on glioblastoma cell lines. In the latter instance, the plasma discharge from a 70 µm device increased ROS and RNS levels and this resulted in marked reduction in U87MG glioblastoma cell viability. Most importantly, this device was also able to apply plasma discharge through an intracranial endoscopic tube to mice brains, which resulted in tumour growth suppression [125]. The authors then optimised the device parameters for the most suitable treatment of brain and breast tumours [126].

The in vivo plasma effect has been demonstrated in animal models subcutaneously injected with 4T breast cell lines. The mouse body tumour growths from these cell lines were exposed to plasma discharge from a 250 µm diameter CAP device. Importantly, plasma administration for 3 min achieved tumour growth reduction comparable with chemotherapy. There was also significant change in the ratio of pro- and anti-apoptotic gene activity at the molecular level [127].

Mashayekh et al. [128] then studied the in vivo effect of CAP on mouse models and the in vitro influence on melanone B16/F10 cell lines. Their work demonstrated lost viability in most cell lines and marked shrinkage of the animal model tumours. The cell-line results were achieved in 48 h after 3 min CAP treatment and in vivo tumour shrinkage was comparable to that achieved by chemotherapy. In summary, analyses of CAP effects on skin tumour treatments were the first performed because plasma is most easily applied directly to dermal structures. For example, G361 melanoma cells lost viability and detached from the surface after CAP application. These cells had lower integrin and FAK expression and altered actin filament structure. This result supports the hypothesis that plasma-facilitated cell death may be related to integrin–ECM interactions [129]. It was also possible to selectively increase the CAP antiproliferative effect on melanoma cell lines using plasma with gold nanoparticles bound to the anti-FAK antibodies [130].

MCF-7 breast cancer cell lines were also subjected to analyses. The initial results indicated reduced cell viability after CAP treatment due to increased apoptosis [22]. Ninomiya et al. [131] then demonstrated that CAP induced injury to 50% of cancerous breast cell lines, regardless of whether they were invasive MB-231 or non-invasive MCF-7 cell lines. Finally, CAP antiproliferative effects were also noted in cell lines derived from human breast cell metastases [131].

Further research was conducted on the HCT-116, SW480, and LoVo colon cancer cell lines. These cells lost viability after CAP application [132], and this effect was accompanied by decreased cell mobility and a higher degree of B-catenin phosphorylation. In addition, although the CAP device was considered to promote apoptosis in up to 70% of B16 and COLO320 colorectal cell lines, there was no effect on the macrophage cell line controls [133]. Ishaq et al. [134] then reported that CAP treatment increased apoptosis in various colorectal cell lines through activation of the Nox2-AKT1 pathway. The authors also demonstrated that although HT29 cell lines are largely resistant to ROS-mediated cancer death, this resistance can be reduced by inhibiting Nrf2/Srx signalling. Finally, CAP exposure has antiproliferative effects on multicellular tumour spheroids, which should mimic the tumour microenvironment in a dose-dependent manner. The loss of spheroid Ki67 and accumulated DNA damage were observed following this CAP exposure [135].

In vitro experimental results also suggest that CAP application should benefit neck and head cancers. While tumours in this area are relatively easier to remove, this procedure is often invasive and CAP should reduce this effect. Guerrero-Preston et al. [136] found that CAP also acted in an antiproliferative manner on these cells. Although these authors considered that this resulted from non-apoptotic processes, contrasting studies suggested that the antiproliferative effect emanated from apoptotic cascade activation [137,138].

Several studies have also assessed CAP application on cervix uteri tumours, primarily on HeLa cells. Apoptotic processes in these cells were observed after CAP treatment due to increased ROS and subsequent changes in the JNK and p38 pathways [139]. A further observed mechanism for this effect was that membrane lipid oxidation caused cell collapse [140]. In addition, Tan et al. [141] employed a microdevice with approximately 1 µm electrode diameter and this selectively induced apoptosis in particular HeLa cells without affecting neighbouring cells.

The possibility of CAP device application to leukaemia cell lines has also been assessed. However, the hypothetical application of CAP in clinical conditions remains unclear, and establishing the best method for its application requires further investigation. Here, the use of CAP-activated liquids could also be considered. However, CAP induced in vitro cell death in THP-1 leukaemia cell lines in a dose dependent manner [142]. CAP application also caused uncontrolled necrotic cell death [143]. The authors then established induced cell apoptosis 45 s after exposure to CAP, and necrosis was observed after treatment for more than 50 s. Finally, two independent studies also demonstrated antiproliferative effects on pancreatic cells [144,145].

CAP treatment has also been directly applied to human tumours in clinical settings [146]. This study involved six patients with advanced neck and head cancer. Two of these patients had initial tumour size reduction after CAP application. Although one patient achieved at least partial remission and improvement, the other relapsed and died. Two patients reported no CAP treatment side effects, but the remaining four had increased dry mouth. Most importantly, four patients reported a significant reduction in pain. These patients, however, were in terminal stages, and five had passed away by the end of the study. Although cold plasma application did not reverse the disease course, it had an overall positive effect without significant harm or side effects.

Plasma discharge has been shown to directly and indirectly affect the cell culture medium [25], and this has encouraged consideration of CAP-activated plasma or other liquid use to reduce tumour cell viability. The greatest advantage of plasma-activated medium (PAM), plasma-activated Ringer’s lactate solution (PAL), and other plasma-activated solutions is that they can be stored for a relatively long time. It is also hypothetically believed that activated liquids can be applied to internal structures with relative ease. CAP-activated media generally induce apoptosis due to ROS, RNS, and H_2_O_2_ content [147]. The resultant effect, however, may be cell-specific and dependent on parameters such as the amount of aquaporins in the cell line [148].

Apoptotic morphological changes have been particularly observed in glioblastoma cell lines following CAP-induced media application. Increased effector caspase 3/7 activity and decreased AKT kinase expression have also been observed at the molecular level [122]. Similar results were obtained when gastric cancer cell lines were affected by PAM [149]. These cells began to show morphological changes associated with apoptosis. Caspase 3/7 activity was increased and the proportion of annexin V positive cells in the affected group was significantly higher than in the control group after two hours PAM exposure. The experimental group also had higher ROS, but this could be eliminated by ROS scavengers. Moreover, cells seeded at 1 × 10^4^ per well showed a degree of resistance to PAM, but this subsided after exposure for more than two minutes. There was more highly-expressed CD44 variant 9 in these resistant cells. This variant is important in the synthesis of reduced glutathione, which is necessary for free radical neutralisation and appropriate responses to stress signalling [150].

The cell viability of ovarian cancer cell lines resistant to paclitalex and cis-platin decreased by almost 30% after CAP treatment [151]. The authors also analysed CAP effects on mouse xenografts. Several chemo-resistant cell lines with serum-free medium and matrigel were initially inoculated into mice, and 200 µL of plasma-activated medium and untreated control RPMI-1640 medium were then inoculated. Dependent on cell lines, the tumours decreased by 66% and 52% in 29 days after the first CAP injection compared to control levels. In addition, PAM application to four types of pancreatic tumour lines also increased ROS levels. This led cells to cell apoptosis, which could be reversed by ROS scavengers [145]. The study also assessed the efficiency of PAM on mouse xenografts, and significant tumour mass shrinkage was noted after 28 days. Moreover, authors Nakamura et al. [152] found that in vivo PAM medium exposure to a different mouse xenograft model inhibited peritoneal dissemination in ES2 cancer cells.

In addition to PAM, PAL can also be used for anti-tumour treatment. PAL induces apoptosis under in vitro conditions in pancreatic cell lines by increasing ROS levels and decreasing cell adhesion. PAL also had anti-tumour effect on peritoneal nodules from these cell lines in in vivo mouse xenograft models [153]. In addition, PAL application exerted strong antiproliferative effects on A549 lung cancer cell lines [154]. In that work, CAP application initiated mitochondrial dysfunction connected with downregulation of the NF-κB-Bcl2 molecular pathway [154]. The authors considered that PAL’s final antiproliferative effect on tumour cell lines should be stronger than PAM. This consideration, however, is open to debate and requires further analysis. Regardless of that outcome, PAL’s major advantage is that it will be potentially easier to apply in future clinical trials because currently cultivated media cannot be used in medical treatment [154]. Although this provides an appropriate alternative to current methods, it still requires further study. Finally, analyses of PAM effects on tumour cells should also continue, despite possible complications in its use in clinical trials. These analyses may discover additional phenomena and principles beneficial for clinical procedures.

One of the most advanced uses of CAP is its application in cancer immunotherapy. This is possible because the onset of cancer and its course can be significantly influenced by the human immune system. The immune system’s modulation ability particularly overcomes the cancer cells’ capacity to supress immune responses [155]. These modulations include the use of various cytokines, cell-based therapies, and immune checkpoint blockades [147]. Moreover, some radio- and chemotherapy procedures trigger immunogenic cell death (ICD) [156,157]. Cells damaged or altered by radio- or chemotherapy produce ‘damage-associated molecular pattern signals’ (DMP). The DMP molecules then lead the immune system to destroy those cells. Most importantly, some studies also suggest that CAP induces ICD, and this leads to subsequent macrophage stimulation [38,158,159].

Miller et al. [160] and Almeida [161] described the detailed principles and possibilities of plasma use in immunotherapy. In particular, the work by Lin et al. [162] demonstrated that CAP should induce ICD in vitro in A549 lung cancer and radio-resistant nasopharyngeal carcinoma cell lines. Therein, extracellular ATP secretion was followed by ICD macrophage stimulation. In addition, CAP increased extracellular ROS levels with a resultant increase in calreticulin production. CAP-based immunotherapy is also a promising option for glioblastoma multiforme treatment. This is a very aggressive cancer with immune system downregulation [161]. Finally, Cheng et al. [163] highlighted the following: 1.) 30 s CAP stimulation of macrophages resulted in increased production of IL-6 and IL12 and decreased anti-inflammatory cytokine IL-10 production and 2.) that IL-2 and IFN-g production increased in isolated T-cell lines from mouse spleen following exposure to CAP. The authors attempted to simulate in vivo conditions as they removed mouse model lymph nodes and exposed them to CAP. They then isolated CD4+ T cells from both the control and affected lymph nodes. The cells derived from the affected lymph nodes had increased function, which was manifested in increased production of IL-2 and IFN-g. The authors’ work culminated in showing the strong anti-tumour effect in mice who had CAP-affected T cells transferred to their lymph nodes. Effects of CAP application on variable tumour cell lines are listed in Table 5.

## 7. Conclusions

CAP has significant potential for wide use in modern clinical practice. Analyses already performed indicate that cold plasma application has been beneficial in many areas of medicine, with no significant negative effect on healthy cells. CAP application, however, may produce potentially harmful elements and should therefore be applied under expert guidance and in appropriate dosage. In addition, CAP use should not be limited only to surface structures, because technology advances are likely to enable development of ever-smaller devices capable of applying plasma discharges to internal structures.

These CAP procedures will most likely prove beneficial in the treatment of internal tissues in addition to cancer treatment. However, current analyses of cold plasma effects still focus only on cell lines and animal models. Although these experiments are more accessible and easier to conduct, wider testing of CAP effects in cancer and regenerative medicine studies in human patients is important and should prove beneficial. The potential introduction of CAP in routine clinical practice in regenerative medicine and oncology could significantly reduce the financial burden and costs of these treatments. Most importantly, a CAP treatment regime will be less invasive and stressful for patients.

## Authors Contribution

D.D. and D.B. wrote the original draft; H.Š. and E.H. supervised the project and revised and approved the manuscript. All authors have read and agreed to the published version of the manuscript.

## Figures and Tables

**Table 1:** List of cold atmospheric plasma (CAP) applications for chronic wound healing.

Wound Type	Number of Patients/Subjects	Plasma Device Type/Injected Gas	Result	Exposure Time
Chronic leg ulcers [41]	*n* = 36	MicroPlaSter plasma torch/Ar	Faster wound healing, microbial load reduction	5 min/day
Chronic ulcers [42]	*n* = 24	MicroPlaSter alpha and beta plasma torch/Ar	Faster wound healing, microbial load reduction	2 min/day
Chronic venous leg ulcers [47]	*n* = 14	PlasmaDerm(^®^) VU-2010 DBD device	Strong antimicrobial effect, rapid ulcer size reduction	45 s/cm^2^ (max. 11 min)/3× per week/8 weeks
Chronic pressure ulcers [48]	*n* = 50	Plasma Jet/Ar	Better PUSH score, microbial load reduction	1 min/cm^2^/1× per week/8 weeks
Pyoderma gangrenosum [50]	*n* = 2	Plasma jet device with variable electrode types	Gradual drying, absorbing, and wound healing	>5 min until all area was not irradiated/every second day/6 and 8 times
Chronic leg ulcers due to diabetes [50]	*n* = 1	Plasma jet device with variable electrode types	Gradual healing of wound	>5 min until all area was not irradiated/every second day/3 times
Pressure ulcers in Wistar rats [49]		Plasma jet/He	Rapid re-epithelialisation, angiogenesis, and collagen synthesis	30 s/3× per day/5 days

Ar: Argon, He: Helium.

**Table 2:** List of cold atmospheric plasma (CAP) applications for acute wound healing.

Wound Type	Number of Patients/Subjects	Plasma Device Type/Injected Gas	Result	Exposure Time
Burn wound [64]	*n* = 1	Plasma jet/He	Decrease in pain and itching after first application, re-epithelization after second application	3 min/2 times with 16 h between 1st and 2nd applications
Wounds at the donor skin graft sites [65]	*n* = 40	Plasma jet/Ar	Rapid healing of skin graph donor sites in CAP-treated patients	2 min every day/7 days
CO_2_ laser skin lesion [68]	*n* = 20	kINPen^®^MED plasma jet/Ar	Improved scar recovery, no side effects of plasma demonstrated	3–10 s/3 days
Fractional CO_2_ laser skin wounds [69]	*n* = 12	kINPen MED^®^ plasma jet/Ar	Wound healing effect similar to standardly used treatment, but with reduced redness and mean roughness of the skin	
Traumatic wound [50]	*n* = 2	Plasma jet device with variable electrode types	Stopping of wound exudation, complete wound treatment after three healing procedures	20 min for whole wound/every two days/three repetitions of healing
Wound after genital wart [50]	*n* = 1	Plasma jet device with variable electrode types	Gradual healing of wound	>5 min until all area was not irradiated/every second day/2 times
Burn wounds in animal models [59,60,61,62,63]	*n* = 15 [60], *n* = 12 [62,63]	Plasma jet/He [59], N_2_/Ar [61], Microplasma jet/He [60], Plasma jet/He [62,63]	Anti-inflammatory effect, re-epithelisation, angiogenesis, collagen rearrangement [59,60,61], increased speed of wound healing, changes in biochemical and haematological profile in plasma treated group [62,63]	1–2 min/8 h interval/5 days [60] 40 s per day/21 days [62,63]
Dog bite wound [66]		KinPEN^®^VET plasma jet/Ar	Potential antimicrobial effect on bacterial strains typically presented in dog saliva and dog bite wounds	<2 min of exposition under in vitro conditions

Ar: Argon; He: Helium; N_2_: Nitrogen.

**Table 3:** List of cold atmospheric plasma (CAP) application effects to dental and oral cavity structures under in vitro and ex vivo conditions.

Purpose of CAP Application	Device/Injected Gas	Final Effect	Exposure Time
Dental biofilm reduction [73]	Kinpen MED ^®^ plasma jet/Ar	Antimicrobial effect on *Candida albicans* and *Staphylococcus aureus*, proved no side effects on reconstituted oral epithelium	60 s/sample
Dental biofilm reduction on titanium discs [75]	Three different types of CAP devices: (a) kINPen plasma jet/Ar; (b) HDBD device/Ar; (c) VDBD device/Ar	Antimicrobial effect on wide spectrum of saliva bacterial species, especially *Streptococcus mutans*	1–10 min/sample
Dental canal disinfection [76]	Plasma jet device/Ar/O_2_	Complete reduction of microbial infection in dental canal ex vivo to a depth of 1 mm	5 min/one extracted tooth
Dental canal disinfection [77]	Plasma jet device/He; He/O_2_	Significant reduction of *Enterococcus faecalis* contamination ex vivo, better effectivity when He/O_2_ used as a carrying gas	2–8 min/sample
Improvement of dental structures [80,81,82,85]	Plasma brush/Ar [80]; low-pressure plasma device/O_2_, Ar, N_2_, and He+N_2_ [81]; HDBD device/Ar [82]; plasma jet device [85]	Increased dentin binding [80]; strengthening of links between fibre-reinforced posts and composite core material [81]; increase of hydrophilicity in titanium [82] and zircone [85] structures	30 s/sample [80](62); 10 min [81]; 2–6 min/sample [82]; 30–90 s/sample [85]

Note: Ar: Argon; He: Helium; O_2_: Oxygen; N_2_: Nitrogen, VDBD: Volume dielectric barrier discharge device; HDBD: Hollow dielectric barrier discharge device.

**Table 4:** List of CAP application effects on stem cells and progenitor cells under in vitro conditions.

Type of Cells	Device/Injected Gas	Result	Exposure Time
Stem cells derived from adipose tissues [87]	DBD device/He	2-fold elevated proliferation of stem cells in vitro after CAP treatment, higher levels of NO, higher activity of Akt, ERK1/2, and NF-κB pathways in these cells	50 s per hour/10 times
Osteoprogenitor (MC3T3-E1) cells [88]	DBD discharge NO-plasma nozzle system	Increase of NO in control media and possibility of its introduction into intracellular space, no cytotoxic effects on cells	30–180 s
Osteoprogenitor (MC3T3-E1) cells [89]	DBD NBP device	Decrease in PIK3/AKT and MAP signalling, increase in p38 signalling, dephosphorylation of FOXO1 transcriptional factor	1–10 min
Human mesenchymal stem cells isolated from periodontal ligaments [91]	Plasma needle/He	Reduced migration of cells, loss of adhesivity, osteo-differentiation of these cells	10–120 s
Hematopoietic stem cells; bone marrow-derived stem cells [92]	DBD Device/He	Increased proliferation of cells, higher expression of surface markers CD44 and CD105, higher expression of *Oct4*, *Sox2*, and *Nanog* genes, effect of CAP on G1-S transition	50 s per hour/0 times
Murine neural stem cells (C.17-2.NSC) [94]	Plasma jet He/O_2_	Higher proliferation and differentiation levels of cells, main portion of neural cells differentiated to neurons	60 s
Murine neuroblastoma stem cell (N2a) [99]	DBD device/O_2_+N_2_	Higher proliferation of cells after CAP exposure, Increase of NO induced inhibition of cytochrome c oxidase, activation of Trk/Ras/ERK pathway	1–10 min
Adipose-derived stromal cells [100]	DBD device/He	Senescence phenotype of cells, proliferation arrest of cells, increase in p53/p21 damage, morphological changer typical for changes in p16 activity	3 min/sample, incubation one hour in PAM

He: Helium; O_2_: oxygen; N_2_: nitrogen; NO: nitric oxide; NBP: non-thermal biocompatible plasma; DBD: dielectric barrier discharge; PAM: plasma-activated medium.

**Table 5:** List of cold atmospheric plasma (CAP) in vitro applications for tumour treatment.

Cell Line Type	Plasma Device/Injected Gas	Exposure Time
Melanoma cell lines [114,128,129,130]	Plasma jet/He [114]; Plasma jet device/He [128]; Plasma micro jet/He [129]; DBD device [130]	30 s [114]; 3 min [128]; 15 s [129]; 40 s [130]
Breast cancer cell lines [22,115,131]	Plasma jet [115]; Plasma jet/He+O_2_ [22]; Plasma jet/He [131]	150 s [115]; 5–30 s [22]; >30 s [131]
Cervical cancer cell lines [139,140,141]	Plasma jet [139]; Plasma jet [140]; Plasma microjet [141]	5 min [139]; 10–15 s [141]
Brain tumour cell lines [115,120,121,122,123,125] ^c^	Plasma jet [115]; Surface micro discharge device (hybrid device) [120]; FE-DBD device [121]; NEAPP device [122]; DBD device [123]; Microplasma jet device/He [125]	150 s [115]; 30–120 s [120]; several minutes of medium treatment [122]; 4 min [123]; 5–120 s [125]
Colorectal cancer cell lines [121,132] ^b^ [133,134]	FE-DBD device [121]; Plasma torch/He+O_2_ [132]; Plasma jet/He and Ar [133]; Plasma jet/He [134]	1–4 s [132]; 60–120 s [133]; 5–30 s [134];
Gastric cell lines [149] ^b^	NEAPP jet device/Ar	5 min
Lung cancer cell lines [124] ^a^ [154] ^c^	Microplasma jet device/He [124]; Plasma jet/Ar [154]	3 min of solution treatment [154]
Ovarian cancer cell lines [151] ^b^ [152] ^b^	NEAPP jet device/Ar [151]; NEAPP jet device/Ar	30–300 s [151]; 10 min PAM treatment [152]
Head and neck cancer cell lines [136,137,138]	Plasma jet/He [136]; Plasma jet/He+O_2_ [137]; Kinpen ^®^ MED [138]	10–45 s [136]; 20–150 s [138]
Leukaemia cell lines [142,143]	Plasma pencil/He [142]	10 s–10 min [142]
Pancreatic cell lines [144,145] ^b^ [153] ^c^	Plasma gun/He [144]; Plasma jet/Ar [145]; Plasma jet/Ar [153]	10–90 s [144]; 30 s–5 min [145]; 3 min of liquid treatment [153]

(a) Animal cell lines; (b) Plasma-activated medium; (c) Plasma-activated solution; He: Helium; Ar: Argon; O_2_: oxygen; DBD: dielectric barrier discharge; FE-DBD: floating electrode dielectric barrier discharge; NEAPP: non-equilibrium atmospheric pressure plasma.

## Abbreviations

Ar	Argon
ASC	Adipose tissue stemm cells
CAP	Cold atmospheric plasma
CO_2_	Carbon dioxide
DBD	Dielectric barrier discharge
DMP	Damage-associated molecular pattern signals
FE-DBD	Floating electrode dielectric barrier discharge
He	Helium
H_2_O_2_	Hydrogen peroxide
hPDL– MSC	Stem cells isolated from periodontal ligaments
ICD	Immunogenic cell death
K	Kelvin
NO	Nitric oxide
NO_2_	Nitrogrn dioxide
NO_3_	Nitrogen trioxide
N_2_O	Nitrous oxide
N_2_O_4_	Dinitrogen tetraoxide
NEAPP	Non-equilibrium atmospheric pressure plasma
NBP	Non-thermal biocompatible plasma
NSC	Neural stem cell
N_2_	Nitrogen
OH	Hydroxyl
O_2_	Oxygen
O_3_	Ozone
PAL	Plasma-activated Ringer’s lactate
PAM	Plasma-activated medium
RNS	Reactive nitrogen species
ROS	Reactive oxygen species
SEM	Scanning electron microscope

## References

[1] Langmuir I. (1928). Oscillations in Ionized Gases. Proc. Natl. Acad. Sci. USA.

[2] Lee H.W., Park G.Y., Seo Y.S., Im Y.H., Shim S.B., Lee H.J. (2011). Modelling of Atmospheric Pressure Plasmas for Biomedical Applications. J. Phys. D.

[3] Izadjoo M., Zack S., Kim H., Skiba J. (2018). Medical Applications of Cold Atmospheric Plasma: State of the Science. J. Wound Care.

[4] Bernhardt T., Semmler M.L., Schäfer M., Bekeschus S., Emmert S., Boeckmann L. (2019). Plasma Medicine: Applications of Cold Atmospheric Pressure Plasma in Dermatology. Oxidative Med. Cell. Longev..

[5] Adhikari B.R., Khanal R. (2013). Introduction to the Plasma State of Matter. Himal. Phys..

[6] Bittencourt J.A. (2004). Fundamentals of Plasma Physics.

[7] Chaudhary K., Imam A.M., Rizvi S.Z.H., Ali J. (2018). Plasma Kinetic Theory. Kinetic Theory.

[8] Sakudo A., Yagyu Y., Onodera T. (2019). Disinfection and Sterilization Using Plasma Technology: Fundamentals and Future Perspectives for Biological Applications. Int. J. Mol. Sci..

[9] Conrads H., Schmidt M. (2000). Plasma Generation and Plasma Sources. Plasma Sources Sci. Technol..

[10] Fridman G., Friedman G., Gutsol A., Shekhter A.B., Vasilets V.N., Fridman A. (2008). Applied Plasma Medicine. Plasma Process. Polym..

[11] Fiebrandt M., Lackmann J.W., Stapelmann K. (2018). From Patent to Product? 50 Years of Low-Pressure Plasma Sterilization. Plasma Process. Polym..

[12] Laroussi M. (2018). Plasma Medicine: A Brief Introduction. Plasma.

[13] Napp J., Daeschlein G., Napp M., von Podewils S., Gümbel D., Spitzmueller R., Fornaciari P., Hinz P., Jünger M. (2015). On the History of Plasma Treatment and Comparison of Microbiostatic Efficacy of a Historical High-Frequency Plasma Device with Two Modern Devices. GMS Hyg. Infect. Control.

[14] Klämpfl T.G., Isbary G., Shimizu T., Li Y.F., Zimmermann J.L., Stolz W., Schlegel J., Morfill G.E., Schmidt H.U. (2012). Cold Atmospheric Air Plasma Sterilization against Spores and Other Microorganisms of Clinical Interest. Appl. Environ. Microbiol..

[15] Lu H., Patil S., Keener K.M., Cullen P.J., Bourke P. (2014). Bacterial Inactivation by High-Voltage Atmospheric Cold Plasma: Influence of Process Parameters and Effects on Cell Leakage and DNA. J. Appl. Microbiol..

[16] Graves D.B. (2014). Reactive Species from Cold Atmospheric Plasma: Implications for Cancer Therapy. Plasma Process. Polym..

[17] Yan D., Sherman J.H., Keidar M. (2017). Cold Atmospheric Plasma, a Novel Promising Anti-Cancer Treatment Modality. Oncotarget.

[18] Pai K., Timmons C., Roehm K.D., Ngo A., Narayanan S.S., Ramachandran A., Jacob J.D., Ma L.M., Madihally S.V. (2018). Investigation of the Roles of Plasma Species Generated by Surface Dielectric Barrier Discharge. Sci. Rep..

[19] Kim S.J., Chung T.H. (2016). Cold Atmospheric Plasma Jet-Generated RONS and Their Selective Effects on Normal and Carcinoma Cells. Sci. Rep..

[20] Kalghatgi S., Kelly C.M., Cerchar E., Torabi B., Alekseev O., Fridman A., Friedman G., Azizkhan-Clifford J. (2011). Effects of Non-Thermal Plasma on Mammalian Cells. PLoS ONE.

[21] Thiyagarajan M., Anderson H., Gonzales X.F. (2014). Induction of Apoptosis in Human Myeloid Leukemia Cells by Remote Exposure of Resistive Barrier Cold Plasma. Biotechnol. Bioeng..

[22] Kim S.J., Chung T.H., Bae S.H., Leem S.H. (2010). Induction of Apoptosis in Human Breast Cancer Cells by a Pulsed Atmospheric Pressure Plasma Jet. Appl. Phys. Lett..

[23] Von Woedtke T., Schmidt A., Bekeschus S., Wende K., Weltmann K.D. (2019). Plasma Medicine: A Field of Applied Redox Biology. In Vivo.

[24] Takamatsu T., Uehara K., Sasaki Y., Miyahara H., Matsumura Y., Iwasawa A., Ito N., Azuma T., Kohno M., Okino A. (2014). Investigation of Reactive Species Using Various Gas Plasmas. RSC Adv..

[25] Tanaka H., Nakamura K., Mizuno M., Ishikawa K., Takeda K., Kajiyama H., Utsumi F., Kikkawa F., Hori M. (2016). Non-Thermal Atmospheric Pressure Plasma Activates Lactate in Ringer’s Solution for Anti-Tumor Effects. Sci. Rep..

[26] Xiong Z. (2018). Cold Atmospheric Pressure Plasmas (CAPs) for Skin Wound Healing. Plasma Medicine-Concepts and Clinical Applications.

[27] Von Woedtke T., Reuter S., Masur K., Weltmann K.D. (2013). Plasmas for Medicine. Phys. Rep..

[28] Keidar M., Shashurin A., Volotskova O., Stepp M.A., Srinivasan P., Homepage J., Sandler A., Trink B. (2013). Cold Atmospheric Plasma in Cancer Therapy Additional Information on Phys. Plasmas Cold Atmospheric Plasma in Cancer Therapy A). Cit. Phys. Plasmas.

[29] Nastuta A.V., Pohoata V., Topala I. (2013). Atmospheric Pressure Plasma Jet-Living Tissue Interface: Electrical, Optical, and Spectral Characterization. J. Appl. Phys..

[30] Gentile R.D. (2018). Cool Atmospheric Plasma (J-Plasma) and New Options for Facial Contouring and Skin Rejuvenation of the Heavy Face and Neck. Facial Plast. Surg..

[31] Chutsirimongkol C., Boonyawan D., Polnikorn N., Techawatthanawisan W., Kundilokchai T. (2014). Non-Thermal Plasma for Acne Treatment and Aesthetic Skin Improvement. Plasma Med..

[32] Bogle M.A., Arndt K.A., Dover J.S. (2007). Evaluation of Plasma Skin Regeneration Technology in Low-Energy Full-Facial Rejuvenation. Arch. Dermatol..

[33] Isbary G., Shimizu T., Li Y.F., Stolz W., Thomas H.M., Morfill G.E., Zimmermann J.L. (2013). Cold Atmospheric Plasma Devices for Medical Issues. Expert Rev. Med. Devices.

[34] Hoffmann C., Berganza C., Zhang J. (2013). Cold Atmospheric Plasma: Methods of Production and Application in Dentistry and Oncology. Med. Gas Res..

[35] Brandenburg R. (2017). Dielectric Barrier Discharges: Progress on Plasma Sources and on the Understanding of Regimes and Single Filaments. Plasma Sources Sci. Technol..

[36] Zhang S., Chen Z., Zhang B., Chen Y. (2019). Numerical Investigation on the Effects of Dielectric Barrier on a Nanosecond Pulsed Surface Dielectric Barrier Discharge. Molecules.

[37] Voráč J., Synek P., Procházka V., Hoder T. (2017). State-by-State Emission Spectra Fitting for Non-Equilibrium Plasmas: OH Spectra of Surface Barrier Discharge at Argon/Water Interface. J. Phys. D Appl. Phys..

[38] Azzariti A., Iacobazzi R.M., Di Fonte R., Porcelli L., Gristina R., Favia P., Fracassi F., Trizio I., Silvestris N., Guida G. (2019). Plasma-Activated Medium Triggers Cell Death and the Presentation of Immune Activating Danger Signals in Melanoma and Pancreatic Cancer Cells. Sci. Rep..

[39] Bauer G., Sersenová D., Graves D.B., Machala Z. (2019). Cold Atmospheric Plasma and Plasma-Activated Medium Trigger RONS-Based Tumor Cell Apoptosis. Sci. Rep..

[40] Kilmer S., Semchyshyn N., Shah G., Fitzpatrick R. (2007). A Pilot Study on the Use of a Plasma Skin Regeneration Device (Portrait^®^ PSR3) in Full Facial Rejuvenation Procedures. Lasers Med. Sci..

[41] Isbary G., Morfill G., Schmidt H.U., Georgi M., Ramrath K., Heinlin J., Karrer S., Landthaler M., Shimizu T., Steffes B. (2010). A First Prospective Randomized Controlled Trial to Decrease Bacterial Load Using Cold Atmospheric Argon Plasma on Chronic Wounds in Patients. Br. J. Dermatol..

[42] Isbary G., Heinlin J., Shimizu T., Zimmermann J.L., Morfill G., Schmidt H.U., Monetti R., Steffes B., Bunk W., Li Y. (2012). Successful and Safe Use of 2 Min Cold Atmospheric Argon Plasma in Chronic Wounds: Results of a Randomized Controlled Trial. Br. J. Dermatol..

[43] Scotton M.F., Miot H.A., Abbade L.P.F. (2014). Factors That Influence Healing of Chronic Venous Leg Ulcers: A Retrospective Cohort. An. Bras. Dermatol..

[44] Bevis P., Earnshaw J. (2011). Venous Ulcer RE. Clin. Cosmet. Investig. Dermatol..

[45] Zmudzińska M., Czarnecka-Operacz M., Silny W. (2005). Analysis of Antibiotic Susceptibility and Resistance of Leg Ulcer Bacterial Flora in Patients Hospitalized at Dermatology Department, Poznań University Hospital. Acta Dermatovenerol. Croat..

[46] Rit K., Sarkar A., Maiti P., Nag F. (2013). Chronic Venous Leg Ulcer with Multidrug Resistant Bacterial Infection in a Tertiary Care Hospital of Eastern India. J. Sci. Soc..

[47] Brehmer F., Haenssle H.A., Daeschlein G., Ahmed R., Pfeiffer S., Görlitz A., Simon D., Schön M.P., Wandke D., Emmert S. (2015). Alleviation of Chronic Venous Leg Ulcers with a Hand-Held Dielectric Barrier Discharge Plasma Generator (PlasmaDerm ^®^ VU-2010): Results of a Monocentric, Two-Armed, Open, Prospective, Randomized and Controlled Trial (NCT01415622). J. Eur. Acad. Dermatol. Venereol..

[48] Chuangsuwanich A., Assadamongkol T., Boonyawan D. (2016). The Healing Effect of Low-Temperature Atmospheric-Pressure Plasma in Pressure Ulcer: A Randomized Controlled Trial. Int. J. Low. Extrem. Wounds.

[49] Chatraie M., Torkaman G., Khani M., Salehi H., Shokri B. (2018). In Vivo Study of Non-Invasive Effects of Non-Thermal Plasma in Pressure Ulcer Treatment. Sci. Rep..

[50] Gao J., Wang L., Xia C., Yang X., Cao Z., Zheng L., Ko R., Shen C., Yang C., Cheng C. (2019). Cold Atmospheric Plasma Promotes Different Types of Superficial Skin Erosion Wounds Healing. Int. Wound J..

[51] Klebes M., Ulrich C., Kluschke F., Patzelt A., Vandersee S., Richter H., Bob A., von Hutten J., Krediet J.T., Kramer A. (2015). Combined Antibacterial Effects of Tissue-Tolerable Plasma and a Modern Conventional Liquid Antiseptic on Chronic Wound Treatment. J. Biophotonics.

[52] Ulrich C., Kluschke F., Patzelt A., Vandersee S., Czaika V.A., Richter H., Bob A., Von Hutten J., Painsi C., Hügel R. (2015). Clinical Use of Cold Atmospheric Pressure Argon Plasma in Chronic Leg Ulcers: A Pilot Study. J. Wound Care.

[53] Schmidt A., Bekeschus S., Wende K., Vollmar B., von Woedtke T. (2017). A Cold Plasma Jet Accelerates Wound Healing in a Murine Model of Full-Thickness Skin Wounds. Exp. Dermatol..

[54] Haertel B., Wende K., Von Woedtke T., Weltmann K.D., Lindequist U. (2011). Non-Thermal Atmospheric-Pressure Plasma Can Influence Cell Adhesion Molecules on HaCaT-Keratinocytes. Exp. Dermatol..

[55] Schmidt A., Von Woedtke T., Sander B. (2016). Periodic Exposure of Keratinocytes to Cold Physical Plasma: An In Vitro Model for Redox-Related Diseases of the Skin. Oxidative Med. Cell. Longev..

[56] Schmidt A., Bekeschus S., Jarick K., Hasse S., Von Woedtke T., Wende K. (2019). Cold Physical Plasma Modulates P53 and Mitogen-Activated Protein Kinase Signaling in Keratinocytes. Oxid. Med. Cell. Longev..

[57] Shome D., von Woedtke T., Riedel K., Masur K. (2020). The HIPPO Transducer YAP and Its Targets CTGF and Cyr61 Drive a Paracrine Signalling in Cold Atmospheric Plasma-Mediated Wound Healing. Oxid. Med. Cell. Longev..

[58] Zhao B., Ye X., Yu J., Li L., Li W., Li S., Yu J., Lin J.D., Wang C.Y., Chinnaiyan A.M. (2008). TEAD Mediates YAP-Dependent Gene Induction and Growth Control. Genes Dev..

[59] Duchesne C., Banzet S., Lataillade J., Rousseau A., Frescaline N. (2019). Cold Atmospheric Plasma Modulates Endothelial Nitric Oxide Synthase Signalling and Enhances Burn Wound Neovascularisation. J. Pathol..

[60] Lee O.J., Ju H.W., Khang G., Sun P.P., Rivera J., Cho J.H., Park S.J., Eden J.G., Park C.H. (2016). An Experimental Burn Wound-Healing Study of Non-Thermal Atmospheric Pressure Microplasma Jet Arrays. J. Tissue Eng. Regen. Med..

[61] Ngo Thi M.H., Shao P.L., Der Liao J., Lin C.C.K., Yip H.K. (2014). Enhancement of Angiogenesis and Epithelialization Processes in Mice with Burn Wounds through ROS/RNS Signals Generated by Non-Thermal N2/Ar Micro-Plasma. Plasma Process. Polym..

[62] Nastuta A.V., Pohoata V., Vasile Nastuta A., Topala I., Grigoras C., Popa G. (2011). Stimulation of Wound Healing by Helium Atmospheric Pressure Plasma Treatment. Artic. J. Phys. D Appl. Phys..

[63] Topala I., Nastuta A. (2012). Helium Atmospheric Pressure Plasma Jet: Diagnostics and Application for Burned Wounds Healing. NATO Science for Peace and Security Series A: Chemistry and Biology.

[64] Betancourt-Ángeles M., Peña-Eguiluz R., López-Callejas R., Domínguez-Cadena N.A., Mercado-Cabrera A., Muñoz-Infante J., Rodríguez-Méndez B.G., Valencia-Alvarado R., Moreno-Tapia J.A. (2017). Treatment in the Healing of Burns with a Cold Plasma Source. Int. J. Burns Trauma.

[65] Heinlin J., Zimmermann J.L., Zeman F., Bunk W., Isbary G., Landthaler M., Maisch T., Monetti R., Morfill G., Shimizu T. (2013). Randomized Placebo-Controlled Human Pilot Study of Cold Atmospheric Argon Plasma on Skin Graft Donor Sites. Wound Repair Regen..

[66] Winter S., Meyer-Lindenberg A., Wolf G., Reese S., Nolff M.C. (2020). In Vitro Evaluation of the Decontamination Effect of Cold Atmospheric Argon Plasma on Selected Bacteria Frequently Encountered in Small Animal Bite Injuries. J. Microbiol. Methods.

[67] Winter S., Nolff M., Reese S., Meyer-Lindenberg A. (2018). Vergleich Der Effizienz von Polyhexanid-Biguanid, Argon-Kaltplasma Und Kochsalzlavage Zur Dekontamination von Bisswunden Beim Hund. Tierärztliche Prax. Ausgabe K Kleintiere / Heimtiere.

[68] Metelmann H.R., Vu T.T., Do H.T., Le T.N.B., Hoang T.H.A., Phi T.T.T., Luong T.M.L., Doan V.T., Nguyen T.T.H., Nguyen T.H.M. (2013). Scar Formation of Laser Skin Lesions after Cold Atmospheric Pressure Plasma (CAP) Treatment: A Clinical Long Term Observation. Clin. Plasma Med..

[69] Nishijima A., Fujimoto T., Hirata T., Nishijima J. (2019). Effects of Cold Atmospheric Pressure Plasma on Accelerating Acute Wound Healing: A Comparative Study among 4 Different Treatment Groups. Mod. Plast. Surg..

[70] Ranjan R., Krishnamraju P.V., Shankar T., Gowd S. (2017). Nonthermal Plasma in Dentistry: An Update. J. Int. Soc. Prev. Community Dent..

[71] Pan J., Sun K., Liang Y., Sun P., Yang X., Wang J., Zhang J., Zhu W., Fang J., Becker K.H. (2013). Cold Plasma Therapy of a Tooth Root Canal Infected with Enterococcus Faecalis Biofilms in Vitro. J. Endod..

[72] Vandana B.L. (2014). From Distant Stars to Dental Chairs: An Update on Plasma Needle. Int. J. Dent. Sci. Res..

[73] Aparecida Delben J., Evelin Zago C., Tyhovych N., Duarte S., Eduardo Vergani C. (2016). Effect of Atmospheric-Pressure Cold Plasma on Pathogenic Oral Biofilms and in Vitro Reconstituted Oral Epithelium. PLoS ONE.

[74] Marsh P.D., Zaura E. (2017). Dental Biofilm: Ecological Interactions in Health and Disease. J. Clin. Periodontol..

[75] Koban I., Holtfreter B., Hübner N.O., Matthes R., Sietmann R., Kindel E., Weltmann K.D., Welk A., Kramer A., Kocher T. (2011). Antimicrobial Efficacy of Non-Thermal Plasma in Comparison to Chlorhexidine against Dental Biofilms on Titanium Discs in Vitro - Proof of Principle Experiment. J. Clin. Periodontol..

[76] Jiang C., Chen M.-T., Gorur A., Schaudinn C., Jaramillo D.E., Costerton J.W., Sedghizadeh P.P., Vernier P.T., Gundersen M.A. (2009). Nanosecond Pulsed Plasma Dental Probe. Plasma Process. Polym..

[77] Armand A., Khani M., Asnaashari M., AliAhmadi A., Shokri B. (2019). Comparison Study of Root Canal Disinfection by Cold Plasma Jet and Photodynamic Therapy. Photodiagnosis Photodyn. Ther..

[78] Wang Q.Q., Zhang C.F., Chu C.H., Zhu X.F. (2012). Prevalence of Enterococcus Faecalis in Saliva and Filled Root Canals of Teeth Associated with Apical Periodontitis. Int. J. Oral Sci..

[79] Shahmohammadi Beni M., Han W., Yu K.N. (2019). Dispersion of OH Radicals in Applications Related to Fear-Free Dentistry Using Cold Plasma. Appl. Sci..

[80] Dong X., Chen M., Wang Y., Yu Q. (2014). A Mechanistic Study of Plasma Treatment Effects on Demineralized Dentin Surfaces for Improved Adhesive/Dentin Interface Bonding. Clin. Plasma Med..

[81] Yavirach P., Chaijareenont P., Boonyawan D., Pattamapun K., Tunma S., Takahashi H., Arksornnukit M. (2009). Effects of Plasma Treatment on the Shear Bond Strength between Fiberreinforced Composite Posts and Resin Composite for Core Build-Up. Dent. Mater. J..

[82] Yang Y., Guo J., Zhou X., Liu Z., Wang C., Wang K., Zhang J., Wang Z. (2018). A Novel Cold Atmospheric Pressure Air Plasma Jet for Peri-Implantitis Treatment: An in Vitro Study. Dent. Mater. J..

[83] Monetto I. (2019). The Effects of an Interlayer Debond on the Flexural Behavior of Three-Layer Beams. Coatings.

[84] Quirynen M., Bollen C.M.L. (1995). CA Novel Cold Atmospheric Pressure Air Plasma Jet for Peri-Implantitis Treatment: An in Vitro Study. J. Clin. Periodontol..

[85] Yang Y., Zheng M., Yang Y., Li J., Su Y.F., Li H.P., Tan J.G. (2020). Inhibition of Bacterial Growth on Zirconia Abutment with a Helium Cold Atmospheric Plasma Jet Treatment. Clin. Oral Investig..

[86] Preissner S., Poehlmann A.C., Schubert A., Lehmann A., Arnold T., Nell O., Rupf S. (2019). Ex Vivo Study Comparing Three Cold Atmospheric Plasma (CAP) Sources for Ebiofllmeremovaleonemicrostructuredetitanium. Plasma Med..

[87] Park J., Lee H., Lee H.J., Kim G.C., Kim D.Y., Han S., Song K. (2016). Non-Thermal Atmospheric Pressure Plasma Efficiently Promotes the Proliferation of Adipose Tissue-Derived Stem Cells by Activating NO-Response Pathways. Sci. Rep..

[88] Elsaadany M., Subramanian G., Ayan H., Yildirim-Ayan E. (2015). Exogenous Nitric Oxide (NO) Generated by NO-Plasma Treatment Modulates Osteoprogenitor Cells Early Differentiation. J. Phys. D Appl. Phys..

[89] Han I., Choi E.H. (2017). The Role of Non-Thermal Atmospheric Pressure Biocompatible Plasma in the Differentiation of Osteoblastic Precursor Cells, MC3T3-E1. Oncotarget.

[90] Klotz L.O., Sánchez-Ramos C., Prieto-Arroyo I., Urbánek P., Steinbrenner H., Monsalve M. (2015). Redox Regulation of FoxO Transcription Factors. Redox Biol..

[91] Miletić M., Mojsilović S., Okićorević I., Maletić D., Puač N., Lazović S., Malović G., Milenković P., Lj Petrović Z., Bugarski D. (2013). Effects of Non-Thermal Atmospheric Plasma on Human Periodontal Ligament Mesenchymal Stem Cells. J. Phys. D Appl. Phys..

[92] Park J., Lee H., Lee H.J., Kim G.C., Kim S.S., Han S., Song K. (2019). Non-Thermal Atmospheric Pressure Plasma Is an Excellent Tool to Activate Proliferation in Various Mesoderm-Derived Human Adult Stem Cells. Free Radic. Biol. Med..

[93] Alemi P.S., Atyabi S.A., Sharifi F., Mohamadali M., Irani S., Bakhshi H., Atyabi S.M. (2019). Synergistic Effect of Pressure Cold Atmospheric Plasma and Carboxymethyl Chitosan to Mesenchymal Stem Cell Differentiation on PCL/CMC Nanofibers for Cartilage Tissue Engineering. Polym. Adv. Technol..

[94] Xiong Z., Zhao S., Yan X. (2019). Nerve Stem Cell Differentiation by a One-Step Cold Atmospheric Plasma Treatment in Vitro. J. Vis. Exp..

[95] Jouhilahti E.M., Peltonen S., Peltonen J. (2008). Class III β-Tubulin Is a Component of the Mitotic Spindle in Multiple Cell Types. J. Histochem. Cytochem..

[96] Foudah D., Monfrini M., Donzelli E., Niada S., Brini A.T., Orciani M., Tredici G., Miloso M. (2014). Expression of Neural Markers by Undifferentiated Mesenchymal-like Stem Cells from Different Sources. J. Immunol. Res..

[97] Weil M.T., Schulz-Ëberlin G., Mukherjee C., Kuo-Elsner W.P., Schäfer I., Müller C., Simons M. (2019). Isolation and Culture of Oligodendrocytes. Methods in Molecular Biology.

[98] Hol E.M., Pekny M. (2015). Glial Fibrillary Acidic Protein (GFAP) and the Astrocyte Intermediate Filament System in Diseases of the Central Nervous System. Curr. Opin. Cell Biol..

[99] Jang J.Y., Hong Y.J., Lim J., Choi J.S., Choi E.H., Kang S., Rhim H. (2018). Cold Atmospheric Plasma (CAP), a Novel Physicochemical Source, Induces Neural Differentiation through Cross-Talk between the Specific RONS Cascade and Trk/Ras/ERK Signaling Pathway. Biomaterials.

[100] Bourdens M., Jeanson Y., Taurand M., Juin N., Carrière A., Clément F., Casteilla L., Bulteau A.L., Planat-Bénard V. (2019). Short Exposure to Cold Atmospheric Plasma Induces Senescence in Human Skin Fibroblasts and Adipose Mesenchymal Stromal Cells. Sci. Rep..

[101] Won H.-R., Kang S.U., Kim H.J., Jang J.Y., Shin Y.S., Kim C.-H. (2018). Non-Thermal Plasma Treated Solution with Potential as a Novel Therapeutic Agent for Nasal Mucosa Regeneration. Sci. Rep..

[102] Scharf C., Eymann C., Emicke P., Bernhardt J., Wilhelm M., Görries F., Winter J., Von Woedtke T., Darm K., Daeschlein G. (2019). Improved Wound Healing of Airway Epithelial Cells Is Mediated by Cold Atmospheric Plasma: A Time Course-Related Proteome Analysis. Hindawi Oxidative Med. Cell. Longev..

[103] Katiyar K.S., Lin A., Fridman A., Keating C.E., Cullen D.K., Miller V. (2019). Non-Thermal Plasma Accelerates Astrocyte Regrowth and Neurite Regeneration Following Physical Trauma in Vitro. Appl. Sci..

[104] Aggarwal V., Tuli H.S., Varol A., Thakral F., Yerer M.B., Sak K., Varol M., Jain A., Khan M.A., Sethi G. (2019). Role of Reactive Oxygen Species in Cancer Progression: Molecular Mechanisms and Recent Advancements. Biomolecules.

[105] Aikman B., De Almeida A., Meier-Menches S.M., Casini A. (2018). Aquaporins in Cancer Development: Opportunities for Bioinorganic Chemistry to Contribute Novel Chemical Probes and Therapeutic Agents. Metallomics.

[106] Yusupov M., Razzokov J., Cordeiro R.M., Bogaerts A. (2019). Transport of Reactive Oxygen and Nitrogen Species across Aquaporin: A Molecular Level Picture. Oxid. Med. Cell. Longev..

[107] Tamma G., Valenti G., Grossini E., Donnini S., Marino A., Marinelli R.A., Calamita G. (2018). Aquaporin Membrane Channels in Oxidative Stress, Cell Signaling, and Aging: Recent Advances and Research Trends. Oxidative Med. Cell. Longev..

[108] Rivel T., Ramseyer C., Yesylevskyy S. (2019). The Asymmetry of Plasma Membranes and Their Cholesterol Content Influence the Uptake of Cisplatin. Sci. Rep..

[109] Van Der Paal J., Neyts E.C., Verlackt C.C.W., Bogaerts A. (2016). Effect of Lipid Peroxidation on Membrane Permeability of Cancer and Normal Cells Subjected to Oxidative Stress. Chem. Sci..

[110] Keidar M., Yan D., Beilis I.I., Trink B., Sherman J.H. (2018). Plasmas for Treating Cancer: Opportunities for Adaptive and Self-Adaptive Approaches. Trends Biotechnol..

[111] Yan D., Talbot A., Nourmohammadi N., Cheng X., Canady J., Sherman J., Keidar M. (2015). Principles of Using Cold Atmospheric Plasma Stimulated Media for Cancer Treatment. Sci. Rep..

[112] Siu A., Volotskova O., Cheng X., Khalsa S.S., Bian K., Murad F., Keidar M., Sherman J.H. (2015). Differential Effects of Cold Atmospheric Plasma in the Treatment of Malignant Glioma. PLoS ONE.

[113] Wiegand C., Fink S., Beier O., Horn K., Pfuch A., Schimanski A., Grünler B., Hipler U.-C., Elsner P. (2016). Dose- and Time-Dependent Cellular Effects of Cold Atmospheric Pressure Plasma Evaluated in 3D Skin Models. Skin Pharmacol. Physiol..

[114] Keidar M., Walk R., Shashurin A., Srinivasan P., Sandler A., Dasgupta S., Ravi R., Guerrero-Preston R., Trink B. (2011). Cold Plasma Selectivity and the Possibility of a Paradigm Shift in Cancer Therapy. Br. J. Cancer.

[115] Kaushik N., Uddin N., Sim G.B., Hong Y.J., Baik K.Y., Kim C.H., Lee S.J., Kaushik N.K., Choi E.H. (2015). Responses of Solid Tumor Cells in DMEM to Reactive Oxygen Species Generated by Non-Thermal Plasma and Chemically Induced ROS Systems. Sci. Rep..

[116] DeBerardinis R.J., Lum J.J., Hatzivassiliou G., Thompson C.B. (2008). The Biology of Cancer: Metabolic Reprogramming Fuels Cell Growth and Proliferation. Cell Metab..

[117] Cha J.-Y., Lee H.-J. (2016). Targeting Lipid Metabolic Reprogramming as Anticancer Therapeutics. J. Cancer Prev..

[118] Ward P.S., Thompson C.B. (2012). Metabolic Reprogramming: A Cancer Hallmark Even Warburg Did Not Anticipate. Cancer Cell.

[119] Xu D., Ning N., Xu Y., Wang B., Cui Q., Liu Z., Wang X., Liu D., Chen H., Kong M.G. (2019). Effect of Cold Atmospheric Plasma Treatment on the Metabolites of Human Leukemia Cells. Cancer Cell Int..

[120] Köritzer J., Boxhammer V., Schäfer A., Shimizu T., Klämpfl T.G., Li Y.-F., Welz C., Schwenk-Zieger S., Morfill G.E., Zimmermann J.L. (2013). Restoration of Sensitivity in Chemo—Resistant Glioma Cells by Cold Atmospheric Plasma. PLoS ONE.

[121] Vandamme M., Robert E., Lerondel S., Sarron V., Ries D., Dozias S., Sobilo J., Gosset D., Kieda C., Legrain B. (2012). ROS Implication in a New Antitumor Strategy Based on Non-Thermal Plasma. Int. J. Cancer.

[122] Tanaka H., Mizuno M., Ishikawa K., Nakamura K., Kajiyama H., Kano H., Kikkawa F., Hori M. (2011). Plasma-Activated Medium Selectively Kills Glioblastoma Brain Tumor Cells by Down-Regulating a Survival Signaling Molecule, AKT Kinase. Plasma Med..

[123] Kaushik N.K., Attri P., Kaushik N., Choi E.H. (2013). A Preliminary Study of the Effect of DBD Plasma and Osmolytes on T98G Brain Cancer and HEK Non-Malignant Cells. Molecules.

[124] Kim J.Y., Ballato J., Foy P., Hawkins T., Wei Y., Li J., Kim S.O. (2011). Apoptosis of Lung Carcinoma Cells Induced by a Flexible Optical Fiber-Based Cold Microplasma. Biosens. Bioelectron..

[125] Chen Z., Simonyan H., Cheng X., Gjika E., Lin L., Canady J., Sherman J.H., Young C., Keidar M. (2017). A Novel Micro Cold Atmospheric Plasma Device for Glioblastoma both in Vitro and in Vivo. Cancers.

[126] Chen Z., Lin L., Zheng Q., Sherman J.H., Canady J., Trink B., Keidar M. (2018). Micro-Sized Cold Atmospheric Plasma Source for Brain and Breast Cancer Treatment. Plasma Med..

[127] Mirpour S., Piroozmand S., Soleimani N., Jalali Faharani N., Ghomi H., Fotovat Eskandari H., Sharifi A.M., Mirpour S., Eftekhari M., Nikkhah M. (2016). Utilizing the Micron Sized Non-Thermal Atmospheric Pressure Plasma inside the Animal Body for the Tumor Treatment Application. Sci. Rep..

[128] Mashayekh S., Rajaee H., Akhlaghi M., Shokri B., Hassan Z.M. (2015). Atmospheric-Pressure Plasma Jet Characterization and Applications on Melanoma Cancer Treatment (B/16-F10). Phys. Plasmas.

[129] Lee H.J., Shon C.H., Kim Y.S., Kim S., Kim G.C., Kong M.G. (2009). Degradation of Adhesion Molecules of G361 Melanoma Cells by a Non-Thermal Atmospheric Pressure Microplasma. New J. Phys..

[130] Kim G.C., Kim G.J., Park S.R., Jeon S.M., Seo H.J., Iza F., Lee J.K. (2009). Air Plasma Coupled with Antibody-Conjugated Nanoparticles: A New Weapon against Cancer. J. Phys. D Appl. Phys..

[131] Ninomiya K., Ishijima T., Imamura M., Yamahara T., Enomoto H., Takahashi K., Tanaka Y., Uesugi Y., Shimizu N. (2013). Evaluation of Extra- and Intracellular OH Radical Generation, Cancer Cell Injury, and Apoptosis Induced by a Non-Thermal Atmospheric-Pressure Plasma Jet. J. Phys. D Appl. Phys..

[132] Kim C.H., Bahn J.H., Lee S.H., Kim G.Y., Jun S.I., Lee K., Baek S.J. (2010). Induction of Cell Growth Arrest by Atmospheric Non-Thermal Plasma in Colorectal Cancer Cells. J. Biotechnol..

[133] Georgescu N., Lupu A.R. (2010). Tumoral and Normal Cells Treatment with High-Voltage Pulsed Cold Atmospheric Plasma Jets. IEEE Trans. Plasma Sci..

[134] Ishaq M., Evans M.D.M., Ostrikov K.K. (2014). Atmospheric Pressure Gas Plasma-Induced Colorectal Cancer Cell Death Is Mediated by Nox2-ASK1 Apoptosis Pathways and Oxidative Stress Is Mitigated by Srx-Nrf2 Anti-Oxidant System. Biochim. Biophys. Acta-Mol. Cell Res..

[135] Plewa J.M., Yousfi M., Frongia C., Eichwald O., Ducommun B., Merbahi N., Lobjois V. (2014). Low-Temperature Plasma-Induced Antiproliferative Effects on Multi-Cellular Tumor Spheroids. New J. Phys..

[136] Guerrero-Preston R., Ogawa T., Uemura M., Shumulinsky G., Valle B.L., Pirini F., Ravi R., Sidransky D., Keidar M., Trink B. (2014). Cold Atmospheric Plasma Treatment Selectively Targets Head and Neck Squamous Cell Carcinoma Cells. Int. J. Mol. Med..

[137] Kang S.U., Cho J.H., Chang J.W., Shin Y.S., Kim K.I., Park J.K., Yang S.S., Lee J.S., Moon E., Lee K. (2014). Nonthermal Plasma Induces Head and Neck Cancer Cell Death: The Potential Involvement of Mitogen-Activated Protein Kinase-Dependent Mitochondrial Reactive Oxygen Species. Cell Death Dis..

[138] Hasse S., Seebauer C., Wende K., Schmidt A., Metelmann H.-R., von Woedtke T., Bekeschus S. (2019). Cold Argon Plasma as Adjuvant Tumour Therapy on Progressive Head and Neck Cancer: A Preclinical Study. Appl. Sci..

[139] Ahn H.J., Kim K., Hoan N.N., Kim C.H., Moon E., Choi K.S., Yang S.S., Lee J.S. (2014). Targeting Cancer Cells with Reactive Oxygen and Nitrogen Species Generated by Atmospheric-Pressure Air Plasma. PLoS ONE.

[140] Kim K., Jun Ahn H., Lee J.H., Kim J.H., Sik Yang S., Lee J.S. (2014). Cellular Membrane Collapse by Atmospheric-Pressure Plasma Jet. Appl. Phys. Lett..

[141] Tan X., Zhao S., Lei Q., Lu X., He G., Ostrikov K. (2014). Single-Cell-Precision Microplasma-Induced Cancer Cell Apoptosis. PLoS ONE.

[142] Barekzi N., Laroussi M. (2012). Dose-Dependent Killing of Leukemia Cells by Low-Temperature Plasma. J. Phys. D Appl. Phys..

[143] Thiyagarajan M., Waldbeser L., Whitmill A. (2012). THP-1 Leukemia Cancer Treatment Using a Portable Plasma Device. Stud. Health Technol. Inform..

[144] Brullé L., Vandamme M., Riès D., Martel E., Robert E., Lerondel S., Trichet V., Richard S., Pouvesle J.M., Le Pape A. (2012). Effects of a Non Thermal Plasma Treatment Alone or in Combination with Gemcitabine in a MIA PaCa2-Luc Orthotopic Pancreatic Carcinoma Model. PLoS ONE.

[145] Hattori N., Yamada S., Tori K., Takeda S., Nakamura K., Tanaka H., Kajiyama H., Kanda M., Fuji T., Nakayama G. (2015). Effectiveness of Plasma Treatment on Pancreatic Cancer Cells. Int. J. Oncol..

[146] Metelmann H.R., Seebauer C., Miller V., Fridman A., Bauer G., Graves D.B., Pouvesle J.M., Rutkowski R., Schuster M., Bekeschus S. (2018). Clinical Experience with Cold Plasma in the Treatment of Locally Advanced Head and Neck Cancer. Clin. Plasma Med..

[147] Tanaka H., Mizuno M., Ishikawa K., Toyokuni S., Kajiyama H., Kikkawa F., Hori M. (2018). New Hopes for Plasma-Based Cancer Treatment. Plasma.

[148] Yan D., Xiao H., Zhu W., Nourmohammadi N., Zhang L.G., Bian K., Keidar M. (2017). The Role of Aquaporins in the Anti-Glioblastoma Capacity of the Cold Plasma-Stimulated Medium. J. Phys. D Appl. Phys..

[149] Torii K., Yamada S., Nakamura K., Tanaka H., Kajiyama H., Tanahashi K., Iwata N., Kanda M., Kobayashi D., Tanaka C. (2015). Effectiveness of Plasma Treatment on Gastric Cancer Cells. Gastric Cancer.

[150] Ishimoto T., Nagano O., Yae T., Tamada M., Motohara T., Oshima H., Oshima M., Ikeda T., Asaba R., Yagi H. (2011). CD44 Variant Regulates Redox Status in Cancer Cells by Stabilizing the XCT Subunit of System Xc- and Thereby Promotes Tumor Growth. Cancer Cell.

[151] Utsumi F., Kajiyama H., Nakamura K., Tanaka H., Mizuno M., Ishikawa K., Kondo H., Kano H., Hori M., Kikkawa F. (2013). Effect of Indirect Nonequilibrium Atmospheric Pressure Plasma on Anti-Proliferative Activity against Chronic Chemo-Resistant Ovarian Cancer Cells In Vitro and In Vivo. PLoS ONE.

[152] Nakamura K., Peng Y., Utsumi F., Tanaka H., Mizuno M., Toyokuni S., Hori M., Kikkawa F., Kajiyama H. (2017). Novel Intraperitoneal Treatment with Non-Thermal Plasma-Activated Medium Inhibits Metastatic Potential of Ovarian Cancer Cells. Sci. Rep..

[153] Sato Y., Yamada S., Takeda S., Hattori N., Nakamura K., Tanaka H., Mizuno M., Hori M., Kodera Y. (2018). Effect of Plasma-Activated Lactated Ringer’s Solution on Pancreatic Cancer Cells In Vitro and In Vivo. Ann. Surg. Oncol..

[154] Matsuzaki T., Kano A., Kamiya T., Hara H., Adachi T. (2018). Enhanced Ability of Plasma-Activated Lactated Ringer’s Solution to Induce A549 cell Injury. Arch. Biochem. Biophys..

[155] Chen D.S., Mellman I. (2017). Elements of Cancer Immunity and the Cancer-Immune Set Point. Nature.

[156] Radogna F., Diederich M. (2018). Stress-Induced Cellular Responses in Immunogenic Cell Death: Implications for Cancer Immunotherapy. Biochem. Pharmacol..

[157] Hernandez C., Huebener P., Schwabe R.F. (2016). Damage-Associated Molecular Patterns in Cancer: A Double-Edged Sword. Oncogene.

[158] Lin A.G., Xiang B., Merlino D.J., Baybutt T.R., Sahu J., Fridman A., Snook A.E., Miller V. (2018). Non-Thermal Plasma Induces Immunogenic Cell Death in Vivo in Murine CT26 Colorectal Tumors. Oncoimmunology.

[159] Khalili M., Daniels L., Lin A., Krebs F.C., Snook A.E., Bekeschus S., Bowne W.B., Miller V. (2019). Non-Thermal Plasma-Induced Immunogenic Cell Death in Cancer. J. Phys. D Appl. Phys..

[160] Miller V., Lin A., Fridman A. (2016). Why Target Immune Cells for Plasma Treatment of Cancer. Plasma Chem. Plasma Process..

[161] Almeida N.D., Klein A.L., Hogan E.A., Terhaar S.J., Kedda J., Uppal P., Sack K., Keidar M., Sherman J.H. (2019). Cold Atmospheric Plasma as an Adjunct to Immunotherapy for Glioblastoma Multiforme. World Neurosurg..

[162] Lin A., Truong B., Patel S., Kaushik N., Choi E.H., Fridman G., Fridman A., Miller V. (2017). Nanosecond-Pulsed DBD Plasma-Generated Reactive Oxygen Species Trigger Immunogenic Cell Death in A549 Lung Carcinoma Cells through Intracellular Oxidative Stress. Int. J. Mol. Sci..

[163] Cheng F., Yan D., Chen J., Keidar M., Sotomayor E. (2019). Cold Plasma with Immunomodulatory Properties Has Significant Anti-Lymphoma Activities in Vitro and In Vivo. Blood.

